# *In silico* mechanistic analysis of IRF3 inactivation and high-risk HPV E6 species-dependent drug response

**DOI:** 10.1038/srep13446

**Published:** 2015-08-20

**Authors:** Masaud Shah, Muhammad Ayaz Anwar, Seolhee Park, Syyada Samra Jafri, Sangdun Choi

**Affiliations:** 1 Department of Molecular Science and Technology, Ajou University, Suwon, 443-749, Korea; 2 The Center of Excellence in Molecular Biology, University of the Punjab, Lahore, 54890, Pakistan

## Abstract

The high-risk human papillomavirus E6 (hrHPV E6) protein has been widely studied due to its implication in cervical cancer. In response to viral threat, activated kinases phosphorylate the IRF3 autoinhibitory domain, inducing type1 interferon production. HPV circumvents the antiviral response through the possible E6 interaction with IRF3 and abrogates p53’s apoptotic activity by recruiting E6-associated protein. However, the molecular mechanism of IRF3 inactivation by hrHPV E6 has not yet been delineated. Therefore, we explored this mechanism through *in silico* examination of protein-protein and protein-ligand docking, binding energy differences, and computational alanine mutagenesis. Our results suggested that the LxxLL motifs of IRF3 binds within the hydrophobic pocket of E6, precluding Ser-patch phosphorylation, necessary for IRF3 activation and interferon induction. This model was further supported by molecular dynamics simulation. Furthermore, protein-ligand docking and drug resistance modeling revealed that the polar patches in the pocket of E6, which are crucial for complex stability and ligand binding, are inconsistent among hrHPV species. Such variabilities pose a risk of treatment failure owing to point mutations that might render drugs ineffective, and allude to multi-drug therapy. Overall, this study reveals a novel perspective of innate immune suppression in HPV infections and suggests a plausible therapeutic intervention.

Every year approximately 0.5 million new cases and nearly 0.25 million deaths occur due to cervical cancer on a worldwide basis. Human papilloma virus (HPV), a small DNA virus, is the leading etiological agent of hyperproliferative lesions and warts in skin, genital organs, and the upper respiratory tract^1^,^2^,^3^. To date, around 15 different species of genus *Alphapapillomavirus* have been characterized^4^,^5^ and nearly one-third of these infect the genital tracts and are transmitted through sexual intercourse^5^,^6^. High-risk HPV (hrHPV) species (HPV16, 18, 26, 31, 33, 34, 35, 39, 45, 51, 52, 56, 58, 59, 66, 68 and 70) act as the etiological agents in 99% of cervical cancers^7^,^8^,^9^,^10^,^11^, whereas HPV16 and 18 and their related types account for 75% and 15% of the total cervical cancer, respectively^12^. Infection by low-risk HPV species (*i.e.*, HPV6, 7, 11, 32, 42, 43, 44, 54, 61, and 71) can cause non-proliferative warts that do not lead to the development of cancer^4^,^13^,^14^.

*E6* and *E7* are two early viral genes that are transcribed into functional proteins after viral entry into cell and are responsible for the cellular transformation and tumorigenesis^15^,^16^,^17^. Genome-wide functional studies have also demonstrated the *in vitro* immortalization of primary human keratinocytes as a result of E6 and E7 expression^18^,^19^. The presence of viral dsRNA in the cell induces interferon regulatory factor 3 (IRF3), which binds to the interferon-β (*IFN-β*) promoter region after forming a stable complex with other transcriptional regulators^20^,^21^,^22^,^23^. Studies have shown that the *IFN-α/β* genes are induced by pathogens that primarily target IRF3^24^, whereas IRF3 targeted by HPV16 E6 protein expressed in cells leads to inhibition of *IFN-β*-mRNA production^25^. This effect is due to HPV16 E6-mediated inhibition of IRF3 transactivation^25^, rather than IRF3 ubiquitination or degradation. Moreover, IRF3-E6 interaction has been studied by Oldak *et al.* and it was found that HPV8-E6, a member of *Betapapillomavirus* genus, did not bind to IRF3 and exhibited a weak antagonizing effect on IRF3 activity^26^. However, tissue tropism and genus-specific interaction of α-HPV E6s to E6-associated protein (E6AP) and β-HPV E6s to mastermind-like 1 (MAML1) protein has been reported, suggesting that this might be due to differences in the LxxLL binding sequence in E6^27^. Modulation of the function of IRF3 by E6 affects cellular immune response^25^, and interaction of E6 with E6AP abrogates apoptosis after the proteaosomal degradation of p53, that enhances the potential oncogenicity of the HPV^28^,^29^,^30^,^31^.

Acidic leucine-rich motifs, such as LxxLL, in IRF3 and E6AP are the primary binding sites of the E6 oncoproteins^25^,^32^. The autoinhibitory domain (AD) flanking the IRF association domain (IAD) maintains IRF3 in an inactive monomeric form in the cytoplasm^33^. In response to viral invasion, the viral induced kinases, IκB kinase (IKK) and TBK (TRAF-associated NFκB activator (TANK)-binding kinase-1), activate IRF3 through phosphorylation^34^,^35^,^36^,^37^,^38^. The activated IRF3 translocates into the nucleus and forms a stable complex with its co-activator, p300/CBP (cAMP response element binding protein), in turn inducing the *IFN-α/β* genes (Fig. 1A).

Activation of IRF3 depends on the kinase binding sites within the C-terminal region of AD. The IRF3 N-terminal and C-terminal segments interact with each other to conceal the H3 and H4 helices in the IAD; the hydrophobic residues in H3 and H4 are involved in DNA binding following AD translocation after IRF3 activation^39^. The monomeric state of IRF3 is maintained by the synergistic activity of the hydrophobic residues in the AD (H1 in the N-terminus and β12, β13, and H5 in the C-terminus). Mutation of a cluster of residues, Ser396 to Ser398 or Ser402 to Ser405, renders IRF3 incapable of undergoing activation and virus-induced phosphorylation^39^. Local structural destabilization is caused by phosphorylation of Ser385, which is located between the IAD and the C-terminal central hinge (loop) region of IRF3^39^. Interaction between the N- and C-termini of the AD structure is likely destabilized by phosphorylation of Ser396 and Ser398. In addition, the minimal phosphoacceptor residue, Ser396, has recently been demonstrated to be responsible for *in vivo* activation of IRF3 in response to viral infection^40^.

To date, the underlying mechanism of hrHPV16 E6 binding to the IRF3-AD has not been elucidated. Structural studies are required to explore the exact mechanism of the transactivation inhibitory activities of E6 toward IRF3 and its subsequent oncogenicity. The interaction between E6 and IRF3 has previously been studied using a yeast two-hybrid system, which demonstrated that IRF3 bound to 55% and 62% of input E6 when truncated at amino acids 149 and 244, respectively^25^. The N-terminus of IRF3 contains two leucine-rich clusters: 140-LDELLG-145 (IRF3-LR1) and 192-LKRLLV-197 (IRF3-LR2), which are postulated as E6 specific binding motifs (Fig. 1B). Using the E6AP-E6 active crystal complex as a control (that contains the E6-binding leucine rich LxxLL motif fused to the maltose-binding periplasmic protein (MBP) (4GIZ))^32^ to validate our protocol, protein-protein docking and molecular dynamics simulations (MDS) were performed to examine the binding affinity of E6 with the aforementioned leucine rich motifs in IRF3. We hypothesized that HPV16 E6 binds to leucine rich motifs of IRF3 in the same manner as it binds to E6AP, thereby rendering it inactive for further phosphorylation by masking the Ser396–398 site. In addition, based on sequence and structure variability, the species-specific drug binding affinity and resistance of E6 were also predicted.

## Results

### Protein-protein docking and MDS

To date, the structural interface between E6 and IRF3 has not been resolved through nuclear magnetic resonance (NMR) or X-ray diffraction studies. To determine the mechanism of E6 binding to IRF3, multiple unrestrained rigid-body docking simulations of the hrHPV E6 protein and of two leucine rich motifs of IRF3 were performed, as described^41^,^42^,^43^. To validate our docking protocol and the binding interface of LxxLL-motifs of IRF3 and E6, the E6AP and E6 chains in the active crystal structure (4GIZ) were separated and then redocked as a control using the online servers (ZDOCK, GRAMM-X, and PyDock). After applying filtering criteria, highly precise results were obtained for the docked conformers, indicating the capability of these servers to reproduce the crystal structure results of E6AP and E6.

To validate and identify the stable residual interface of the docking results, the final selected complexes were subjected to MDS. With respect to the active crystal and the initial docking complexes, the time-dependent RMSDs (root mean squares deviations) of backbone atoms, the distances between the centers of masses of the interacting proteins, and the root mean squares fluctuations (RMSFs) were calculated to assess the conformational stability during the MDS (Supplementary Fig. S1A and B). Based on the average PDB structure, the binding interface of each complex was evaluated. While exploring the E6AP-E6 interface, the binding pattern described in the crystallographic studies^32^ was confirmed (Table 1). In particular, it was found that the leucine-rich motif of the full-length E6AP protein, corresponding to 406-LQELLG-411, plays a crucial role in the interaction with a hydrophobic pocket in E6 (containing Val31, Leu50, Val53, Arg55, Val62, Arg102, and Arg131) formed by two Zn^2^ binding domains (Fig. 2A,B). Domain movements and variation in binding interfaces of the E6AP-E6 crystal along with computationally docked complexes are depicted in 3D animated videos (Supplementary Videos S1 and S2), based on the PCA covariance matrix. The isolated structure of the E6AP-E6 complex had chain folding solvation energies (∆Gf) of −112.7 and −360.8 kcal/mol for E6 and E6AP, respectively. Upon complex formation, E6AP gained −7.9 kcal/mol, while no change was detected for E6. Hydrogen bonding and hydrophobic interactions at the interfaces of E6AP-E6 complexes (crystal and docked) are listed in Tables S1 and S2 in the supporting information.

### E6 binding to leucine rich motifs of IRF3

**Figure 1:**
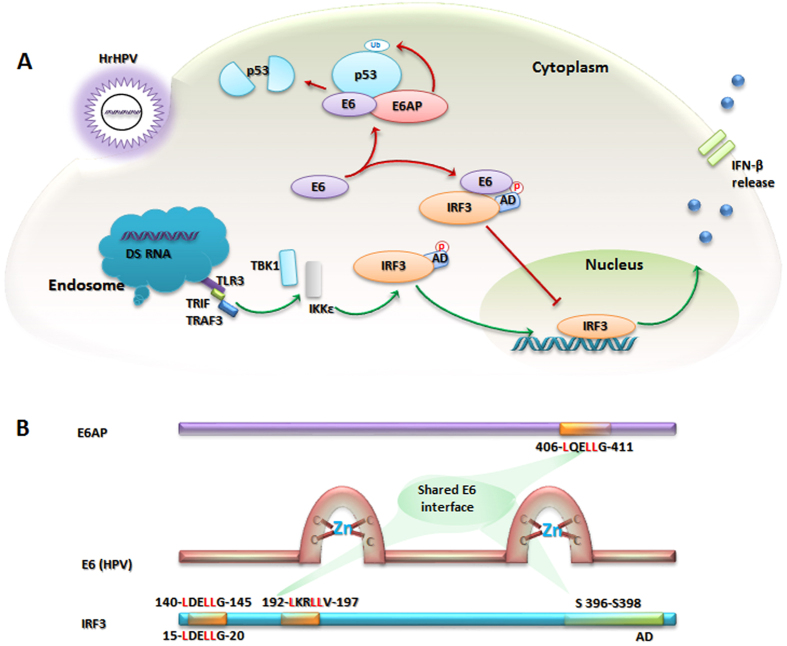
Schematics of E6 binding motifs in IRF3 and E6AP to modulate cell immunity. (**A**) Phosphorylation-dependent transactivation of IRF3 is blocked by E6, thereby limiting the IFN-β based nonspecific antiviral response of cells. E6 recruits E6AP to degrade p53 via the cell proteasome-degradation mechanism after ubiquitination, which disrupts the cell cycle. (**B**) Both IRF3 and E6AP have respective N- and C-termini E6 specific leucine rich motifs that participate in E6 binding. Note: IRF3 residues numbering: above the bar is according to full-length IRF3 (UniProt ID: Q14653, the cyan color bar), while below the bar is according to the 3D model of IRF3-LR1 (leucine rich region 1, 140-LDELLG-145) and this was followed throughout the manuscript.

**Figure 2:**
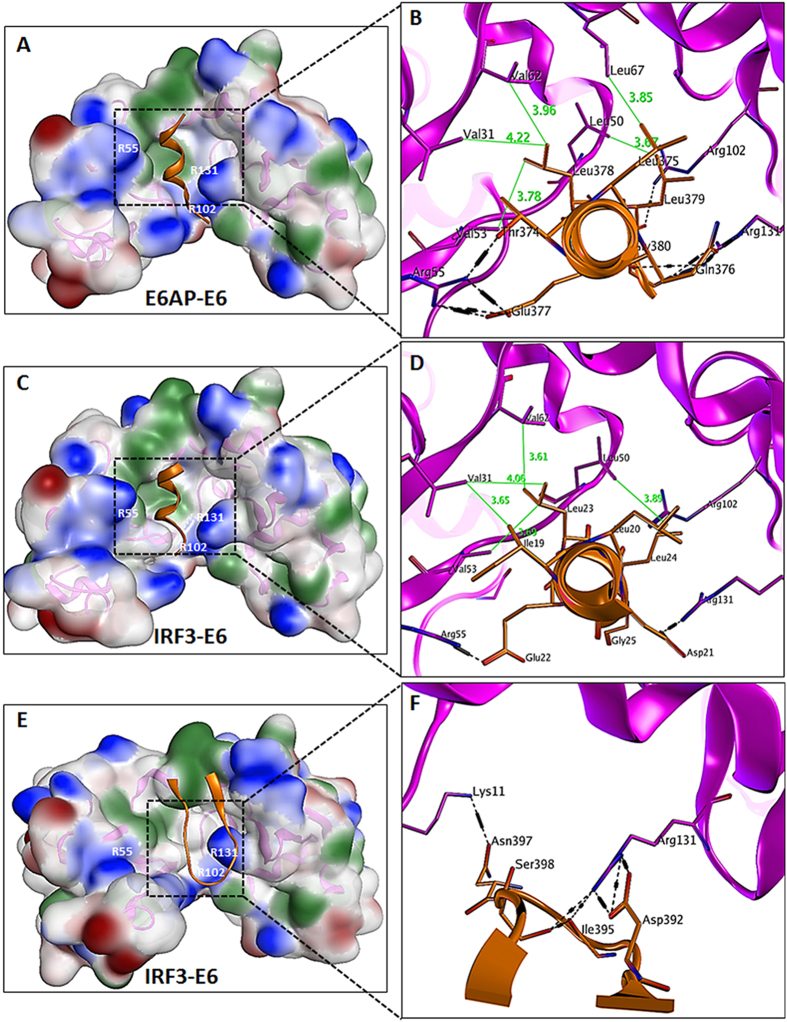
Comparative interface analysis of the E6AP-E6 and E6 binding LxxLL motifs of IRF3. (**A**) Leucine rich motif of E6AP (MBP not shown) bound into the hydrophobic pocket of E6 (residues: green, hydrophobic; blue, positive). The labeled residues are validated through X-ray crystallography by Zanier, *et al.*^32^. (**B**) Arg55, 102, and 131 provide a polar environment for complex stability (blue patches). (**C,D**) IRF3-LR1 (leucine rich region 1) binds into the E6 pocket in similar manner, as E6AP binds. (**E,F**) Binding of the IRF3 autoinhibitory domain (AD) to E6.

Two putative E6 binding leucine rich motifs, IRF3-LR1 and IRF3-LR2, are present in the N-terminal region of IRF3 (Fig. 1B). A 15-mer peptide containing the IRF3-LR1 motif was modeled, while the truncated IRF3 (PDB 1QWT, 189–427) containing LR2 in its AD, was retrieved from PDB and subsequently docked with E6. The 15-mer IRF3-LR1 peptide bound firmly into the hydrophobic pocket of E6 in a similar fashion, as did the E6AP motif, reinforcing our theoretical studies (Fig. 2C,D). When docked as a 15-mer peptide, IRF3-LR2 showed only weak interaction with E6 and was excluded from further evaluations. However, the truncated IRF3 protein was shown to bind to E6 through its AD, which contained only the IRF3-LR2 motif. The Arg194 residue in the IRF3-LR2 motif (LK**R**LLV) disassociated from the binding interface after 40 ns MD simulation, but residues that had been shown to be involved in IRF3 transactivation remained intact (Table 1 and Fig. 2E,F).

### Comparative binding energies of the LxxLL motifs in IRF3 and E6AP

Hot spots in the protein-protein interfaces in the E6AP-E6 and IRF3-E6 docked complexes were identified through the DrugScore^PPI^ server^44^. The binding free energy differences between the wild-type residues and the alanine mutants at hot spots within a protein-protein complex were calculated as follows:
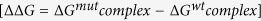


A high and positive ∆∆G value indicates a hot spot with strong binding affinity and vice versa. Comparative binding energy analysis suggested that IRF3-LR1 motifs containing LDELL and E6APs containing the LQELL motif stably bind into the hydrophobic pocket of E6 in a similar way. Leu20, 23, and 24 of LDELL hydrophobically interact within the E6 pocket. In addition to other interactions, the crucial interactions that participate in bringing IRF3 and E6 in close vicinity is formed when the negatively charged residues Asp21 and Glu22 of IRF3 and Arg55 and Arg131 of E6 interact through H-bonds. Similar binding and energy patterns were observed in the E6AP-E6 docked model (Supplementary Fig. S3A & C and Tables S2 & S3). In contrast, the Arg194 residue in the LKRLL motif of IRF3-LR2 initially participated in the interaction, but reduction in its binding energy was observed after MDS. The rest of the interacting residues were primarily those, which are involved in IRF3 transactivation with average binding energies less than 1.5 kcal/mol (Supplementary Fig. S3E and Table S4). The surface map showed that the Ser396 and Ser398-containing loop that transactivates IRF3 marginally interacted with the pocket of E6 (Fig. 2E).

### E6 binds to IRF3-LR1 and hinders the phosphorylation of Ser-patches

**Figure 3:**
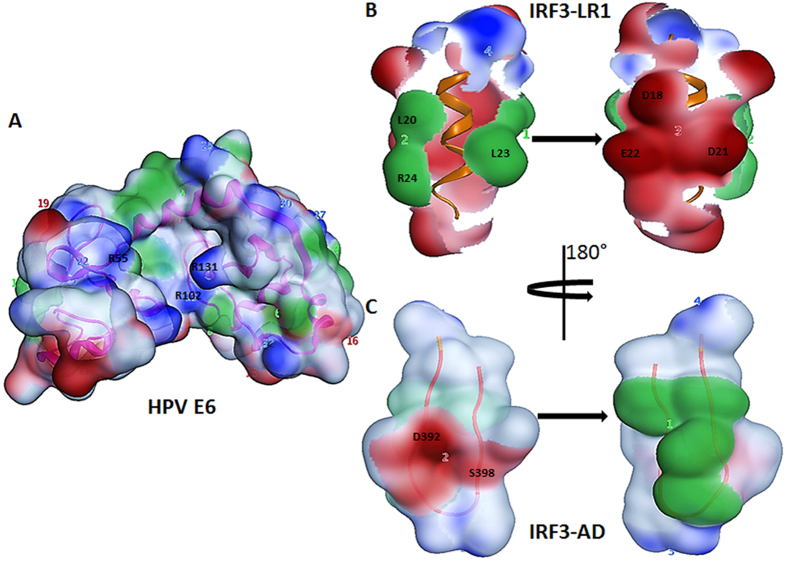
Surface patch representation of the E6 protein and its interaction motifs in IRF3. (**A**) The E6 pocket is dominated by hydrophobic (1, green) and positive (22 and 23, blue) patches. (**B**) Surface patch of IRF3-LR1 represents a lock and key model for E6. The inner surface (patches 1 and 2, green) hydrophobically interacts with the hydrophobic surface of E6 (patch 1) while the outer negative surface (patch 3, red) interacts with the positive patches of E6 (patches 22 and 23). (**C**) The negatively charged serine patch (patch 1) of IRF3-AD interacts with the oppositely charged patches of E6 (patches 22 and 23), which mask it from kinase dependent activation.

The Protein Patch Analyzer distributed in Molecular Operating Environment (MOE; Chemical Computing Group Inc., Montreal, Canada) was used to generate visual representations of protein surface patches as a mean for predicting locations that are highly prone to protein-protein or protein-ligand interactions. As the potential binding mode of E6 had been elucidated, we next proposed that E6 binds to IRF3-LR1 and might interact with the Ser-patch of its AD. As this patch provides a phosphorylation site and is highly valuable for the activation of IRF3, masking it would be expected to render IRF3 unable to be phosphorylated and in turn keeps it inactive. As we have already suggested that E6 binds to the IRF3-LR1 in a similar manner as was observed for E6AP, and secondly E6 also marginally binds to IRF3-LR2. We proposed that besides binding to 140–145 position, E6 also binds to 192–197 region that harbors another LxxLL motif that might account for the increased binding affinity between E6 and IRF3 as previously reported^25^. Our model suggests that IRF-LR2 (192–197) is the possible complementary location for binding, since it is in the transactivation domain, while 140–145 may act as primary binding site to initiate the IRF3-E6 interaction (Fig. 3B). This dual binding may conformationally rearrange the IRF3 protein that might hinder the phosphorylation step at the Ser-patches (Fig. 3C) and is the likely reason why it is not necessary for E6 to either destroy or ubiquinate IRF3 to facilitate viral infection, as it simply precludes its activation. Fig. 2 and Supplementary Fig. S3 illustrate that the binding pockets for both IRF3-LR1 and IRF3-LR2 in E6 share some residues with high binding energy, but the leucine-rich motif in the latter case does not participate in binding; rather, the Asp392, Ile395, Asn397, and Ser398 residues of the IRF3-AD, which are phosphorylated by the virus-induced kinases, actively interact with E6. This provides a possible new mechanism for how hrHPV manages to circumvent the host immune system.

### Ligand binding interface of E6.

**Figure 4:**
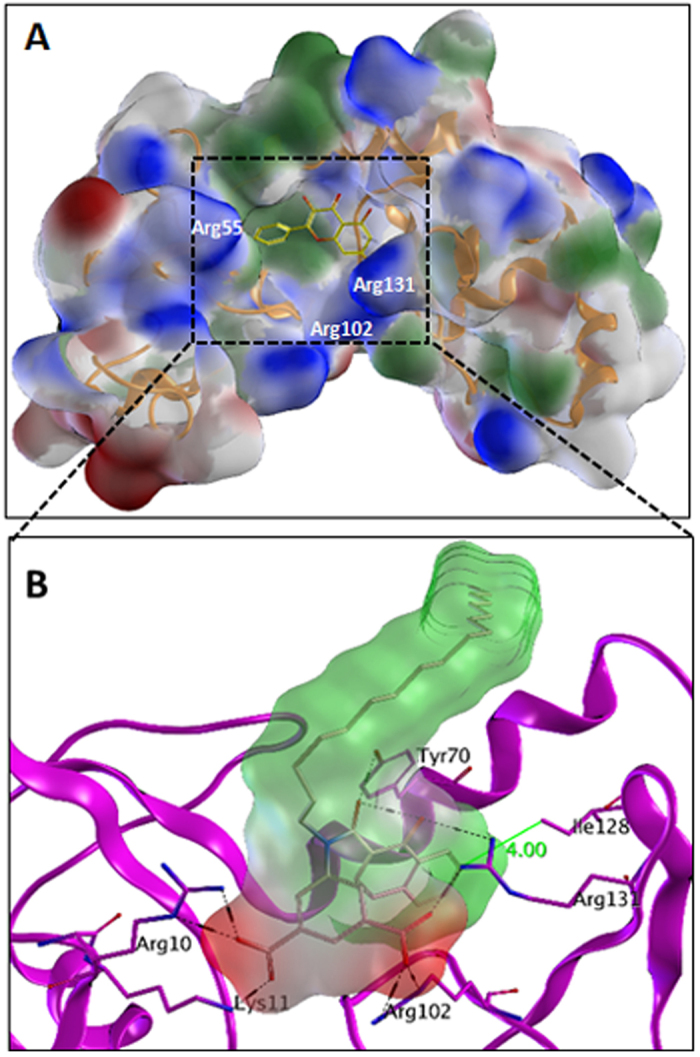
Surface potential and interface analysis of the E6 ligand-bound complex. (**A**) Surface potential of the E6 protein (residues: blue, positive; green, hydrophobic; red, negative). (**B**) The binding position of the ligand interacting with the E6 hot spots present at the complex interface. The hydrophobic aromatic rings of the ligand fit into the hydrophobic pocket of E6, while the negatively charged carboxylic groups (red surface) interact with the positive patches of the pocket.

After exploring the stable interfacial residues of IRF3 and identifying the residues in E6 with high binding energy, the study was extended to find the binding mode of potent E6 inhibitors. The best conformation with the lowest docking score and high binding affinity was selected for each ligand. The docked complexes revealed interacting sites within the hydrophobic pockets of E6 as previously reported^45^,^46^ as well as sites already confirmed in our protein-protein docking results (Fig. 4). The binding affinities of ligands in comparison with their IC50 values are provided in Table 2. The highest binding affinity (pKi = 8.80) was recorded for Morin, a potent E6 inhibitor and a derivative of 5,7-dihydroxy-4H-chromen-4-one, with a hydroxyl group at its *meta* (6′) position on the A′ chain. We expected a direct correlation between the binding affinities and the IC50 values of the reported ligands, but Myricetin and Caf31, with the lowest and highest IC50 values (IC50 = 0.85 and 62.2, respectively) exhibited pKis of 6.74 and 6.44, respectively. Conversely, the topological polar surface areas (TPSAs) of the 5,7-dihydroxy-4H-chromen-4-one and 4H-chromen-4-one derivatives have been correlated with their IC50s (Table 2). Recently, it has been demonstrated that removal of the –OH group from any position in 5,7-dihydroxy-4H-chromen-4-one derivatives results in loss of E6 binding affinity, whereas modification of the surface polarity by substitution of the benzene heterocyclic B-ring (such as with benzoic acid, tetrazole, carboxylic acid, or pthalic acid) can restore ligand activity^46^. To confirm the significance of ligand polarity in the E6 pocket, we mutated the polar arginine residues into alanine and the binding affinity and stability of each complex was determined and are presented in preceding sections.

### MDS of ligand bound E6

To better understand the binding behavior of ligands in the E6 pocket, the E6-ligand complex (SA r278319, Table 2) having the highest binding affinity (8.8 kcal/mol and pKi 8.03) and docking score (−7.36 kcal/mol) was subjected to MDS. With respect to the initial docking complex, the system acquired its stability after a 60 ns production run (Supplementary Fig. S2A), and the distance between the center of the masses remained constant (Supplementary Fig. S2B). Fluctuations were observed in the atoms corresponding to the ligand’s hydrophobic and flexible octadecyl carbon chain; however, the aromatic core portion remained stable (Supplementary Fig. S2C).

A closer look at the interacting residues showed that the positively charged guanidine groups of Arg10, Arg102, Arg129, and Arg131 donated hydrogen bonds to the carboxylic group in the aromatic ring of the ligand. The terminal amide group of Lys11 also interacted with the carboxylic oxygen of the single aromatic ring. The carbonyl oxygen present at the hinge of an aromatic ring accepted the side chain of Tyr70 through a single hydrogen bond. Leu67 and Ile73 made hydrophobic contacts with the carbons of the ring and the octadecyl chain of the ligand (Fig. 4B). Our docking and simulation studies confirmed that the residues of E6 that bound to E6AP and IRF3 with significantly higher binding energies were also engaged by the ligand further reinforcing our proposed IRF3 inactivation model. Furthermore, the results from our study are consistent with the interaction pattern that has been suggested for flavones^46^.

### Principal component analysis (PCA)

**Figure 5:**
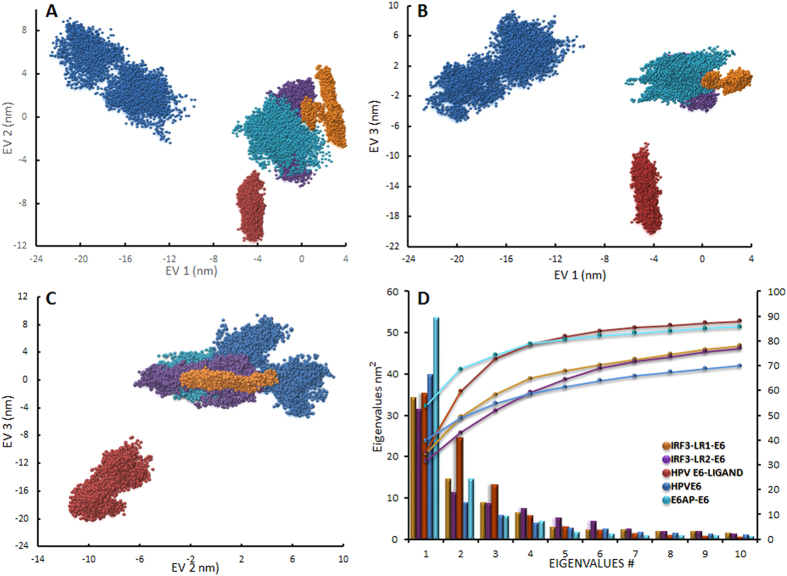
Principal component analyses of different complexes. The cloud represents the projection of trajectories (**A**) eigenvectors (EVs) 1 & 2; (**B**) EVs 1 & 3; and (**C**) EVs 2 & 3. The cyan clouds represent the E6 protein with two distinct minima separated by a transient energy barrier; but when E6 is bound to the ligand, rare or no sampling in other phase space is seen (purple). The green and orange clouds represent E6 bound to E6AP and IRF3, respectively. (**D**) The first 10 EVs of the covariance matrix corresponding to principal motion are represented by bars; the cumulative sum of the contribution to the total fluctuations is represented by lines.

Of major concern for this study was the direction and amplitude of the dominant motions of different domains in the partner proteins in complexes along a simulation trajectory; these can be detected by PCA^47^,^48^. The projections of first three eigenvectors were plotted from five trajectories in the 2D plots, representing the corresponding global motion of E6 alone (dark blue cloud), E6-ligand (red), E6AP-E6 (cyan), and E6 in complex with IRF3-LR1 (orange) and with IRF3-LR2 (purple) (Fig. 5). The eigenvalues for the first ten eigenvectors were plotted, accounting for approximately 70–90% of the prominent characteristic motions of the backbone atoms in all complexes (Fig. 5D). No considerable fluctuations in principal motion and energy transitions were observed for the complexes, but for E6 alone. Removal of the ligand from E6 led to the sampling of the phase space to reach two distinct minima, which were separated by a transit energy barrier. This transition into two different minima is most likely due to the flexible nature of the linker-helix joining the two Zn^+2^ binding domains in E6. When bound to a target protein, the two domains were held together thereby limiting their flexibility and enhancing the complex stability. The directions of motions of different domains of E6 and bound proteins/ligand (i.e., E6AP, truncated IRF3 containing only LR2, 15-mer IRF3-LR1 motif and ligand) are depicted in Supplementary Videos S1-5, constructed through chimera from 60 PDB frames. Prominent motions of the Zn^+2^ binding domains of E6 were observed during MD run. Based on the similar domain movement of E6 in E6AP-E6 and IRF3-LR1-E6 complexes, we suggest that LR1 in IRF3 is the principal binding motif (Supplementary Videos S1 and S3) of E6. However, the shifting of E6 from the LR2 motif to the AD domain (containing Ser396 and Ser398) of IRF3 suggested that E6 might block the transactivation of IRF3 after binding to its LR1 motif (Supplementary Video 4). The wide opening of E6 pocket during MD run suggested the possible anti-E6 potency of reported ligands (Supplementary Video S5).

### Alanine and drug resistance scanning of E6

**Figure 6:**
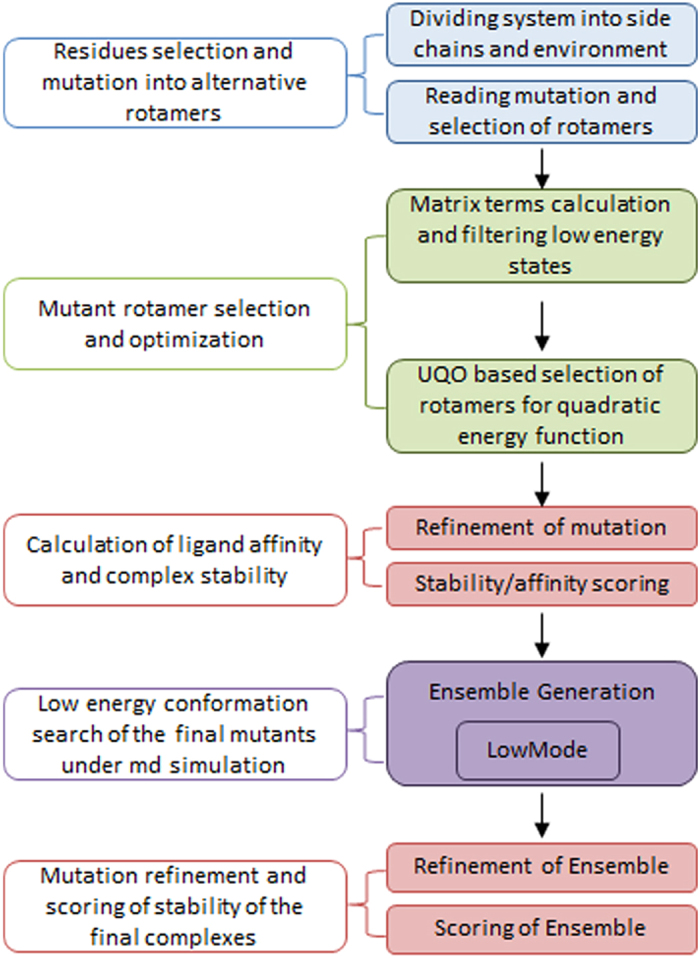
Schematic diagram of alanine and resistance scanning of pocket residues.

A protein design algorithm, illustrated in Fig. 6, was used to look for drug affinities and resistances of the E6 protein. Ligand bound E6 complexes were passed through the described procedure one by one for alanine and resistance scanning. Three residues (Leu50, Arg102, and Arg131), which disrupt LxxLL-E6 binding^32^, were considered for alanine scanning and the resultant changes in ligand binding affinities and complex stabilities were evaluated (Supplementary Fig. S4). Mutation of Arg131 had less effect on ligand binding affinities but greatly contributed to the stability of each ligand-bound E6 complex. This might be the consequence of an increase in solvent-exposed surface area by the replacement of the bulky Arg131 side chain with alanine. On average, no significant loss of ligand affinity was observed when Leu50 was mutated into alanine; however, mutation of Arg102 contributed to a reduction in affinity towards those ligands having attached hydroxyl or carboxylic groups (Supplementary Table S5).

After determining the importance of the above-mentioned residues, the E6 pocket was vetted for possible single nucleotide polymorphisms (SNPs) at each position and their effect on ligand binding affinity. Drug resistance of E6 was determined as a difference in binding affinity of wild type and mutant residues. Using a resistance scan, all mutations specified in the mutation list were created sequentially, and their affinities were calculated (Table S6). A large, positive increase in affinity indicates that the target might easily become resistant to the ligand if the wild type residue is replaced by the respective SNP at that position. Change in relative affinity indicates that E6 might possibly become more resistant to drugs if Arg102 or Arg131 become mutated into uncharged residues.

To determine the conservancy of E6, over 150 full-length amino acid sequences of E6 protein (hrHPV 16, 18, 24, 34, and 53) were downloaded from the UniProt database. A manual filter was applied to remove redundant, 100% identical, and short length sequences. BioEdit^49^ was used to align the sequences and to identify the degree of variability (Supplementary Fig. S5). Based on high sequence similarity, HPV 16 and 18 might possibly behave alike in terms of drug affinity and stability as compared to other hrHPV species of genus *Alphapapillomavirus*. Most of the ligand binding active hot spots in the E6 pocket were found to be conserved in HPV 16 and 18 (with the exception of Arg131), but variations were observed in all other hrHPV species. Resistance scans suggested that SNPs (histidine or methionine) at position Arg131 could lead to drug resistance and might affect the LxxLL binding affinity of E6 as well.

## Discussion

The N-terminal region of IRF3 contains two leucine-rich motifs, *i.e.* 140-LDELLG-145 and 192-LKRLLV-197, that are spatially close to the C-terminus, which contains the Ser396 and Ser398 residues that aid in the relocalization of IAD when it becomes phosphorylated by viral-induced kinases (Fig. 1A). Study using the yeast two-hybrid system has indicated that 55% and 62% of input E6 can bind to the IRF3 when truncated at amino acid 149 and 244 respectively^25^. Interactions of both leucine motifs of IRF3 with E6 have been studied through protein-protein docking simulations followed by extensive computational procedures to predict their binding affinities and specificities. In 15-mer peptide form, the IRF3-LR2 weakly bound to E6, whereas the active crystal of IRF3 containing the IRF3-LR2 motif showed substantial binding with E6, primarily through non-LxxLL residues. Initially, it was found that the Arg194 of the 192-LK**R**LLV-197 motif interacted with the E6 binding pocket, but after MDS, the E6 binding pocket shifted to interact with the AD of IRF3, which is involved in IRF3 transactivation (Supplementary Video S4). In contrast, IRF3-LR1, when docked with E6, remained intact in its hydrophobic pocket for the entire MDS run. The interaction of the DELLG motif (IRF3-LR1) within the E6 binding pocket was also supported by crystallographic study of a structurally and functionally similar leucine rich motif (QELLG) of E6AP, co-crystalized with E6 (Fig. 2A,C)^32^.

To explore the function and binding affinities of important residues, we adopted a computational mutagenesis strategy and calculated the binding free energy of the hot spot residues. Previous analysis of the free energy of decomposition of the E6/LxxLL complex indicated that the E6 Arg102 and Arg131 residues played major roles in van der Wall interactions^32^. Furthermore, mutation of E6 Arg55 and Arg102 residues into alanine decreased E6AP binding and the biological activity of E6^46^. When subjected to alanine scanning, the IRF3-LR1-E6 complex also suggested that Arg55 and Arg131 exhibit high binding energies. Polar patches within the E6 pocket (Arg55, Arg102, and Arg131) substantially affected the stability of the LxxLL-E6 complex when these were mutated into alanine.

It is a common phenomenon that in protein-protein interactions the protein modulation or attenuation is perpetuated. Addition or removal of phosphate or ubiquitin moieties is the prominent and ideal way to alter the functioning of partner proteins. Another mechanism is the masking of active motifs or domains to alter the functions of those proteins. Here, we report a novel demonstration of the latter method to modulate partner protein function, in which E6 binds to IRF3 and might render the phosphorylation site unavailable. This inhibition of kinase-mediated protein activation culminates in the suppression of the immune system as previously reported^50^. Further, a single nucleotide polymorphism (SNP; S427T), which might have caused some structural changes in the IRF3 was significantly related to the persistence of cervical cancer^51^. This and our findings suggest that inhibition of kinase mediated activation or direct inhibition of IRF3 might contribute to reduction in cell’s anti-viral response and progression of cervical cancer.

Effective antiviral agents are still in demand to treat all types of hrHPV infections and to decrease the progression of infection to cervical cancer and the spread of the virus. Recent studies have reported that luteolin and some of its derivatives disrupt the E6AP-E6 interaction by binding within the hydrophobic pocket of E6^46^. In this study, twenty ligands reported to have anti-E6 effects, were computationally docked into the E6 pocket and their binding affinities and behaviors were evaluated through alanine and resistance scanning. The stability of each ligand-bound E6 complex was compromised when Arg131 was mutated into alanine, whereas Arg102Ala mutation reduced the ligand binding affinities. The charged patches and the hydrophobic cavity of the E6 pocket were shown to play a vital role in ligand binding and LxxLL motif recognition. We further wished to identify the effect of any point mutation in the vital residues of E6 on its structural stability and ligand binding affinity. Our findings suggested that the ligand-binding pocket of E6 is not conserved among all hrHPV species and a single ligand effective against one species of HPV might not work against other members; however, based on high sequence similarity, we expect that HPV 16 and 18 species would respond in a similar manner.

In conclusion, our results suggest that hrHPV E6 potentially binds to IRF3 at its leucine-rich motifs and may undergo conformational changes to interact with the Ser-patches, necessary for the activation of IRF3 and induction of interferon. Furthermore, computational analyses suggested that sequence variation in E6 might hamper the efficacy of therapeutic interventions. Therefore, it is extremely necessary to design therapeutic approaches that could withstand minor alteration in E6 sequence and would target the most prevalent hrHPV species. In this regard, this study provides the possible targets validation and furnishes the basis of effective drug design.

## Methods

### Model assessment and protein-protein interaction

The crystals of the interacting partners, IRF3 and E6/E6AP (1QWT and 4GIZ), were retrieved from PDB and analyzed for abnormalities. 4GIZ is a dimer of the MBP-E6AP-E6 complex with missing E6 C-terminal residues (involved in PDZ interaction). The 15-mer N-terminal peptide of IRF3 containing 140-LDELLG-145 was modeled through MOE 2013, using the active crystal structure of E6AP as a template and validated using the protein geometry package distributed within MOE. To check the binding specificity of the 192-LKRLLV-197 domain with E6, a 15-mer peptide was truncated from the crystal structure of IRF3. Unbound solvent and ligand molecules were deleted from all crystal structures. Both IRF3 and E6AP contained E6 specific leucine rich motifs; therefore, the MBP-E6AP-E6 crystal served as a control in our studies to validate the computational protocol used. E6 and E6AP were separated and redocked using three online servers, ZDOCK^52^, GRAMM-X^53^, and PyDock^54^, to validate their docking accuracy by comparing the respective resulting interfaces with the crystalographically defined interface. The same pairwise unrestrained docking protocol was used to determine the interactions between hrHPV E6 and IRF3 motifs.

Based on the electrostatic complementarity, geometry, and hydrophobicity of the molecular surface, GRAMM-X and ZDOCK rank the 100 most probable predictions out of thousands of candidates. PyDock scores the output conformers on the basis of Columbic electrostatics and atomic solvation parameters for rigid-body protein-protein docking under implicit desolvation energy^55^. Furthermore, a three step criterion was defined to filter the docking poses: (i) complexes lacking interaction at the leucine rich motifs (LxxLL) of IRF3 and E6AP with E6 were eliminated; (ii) complexes that agreed with previous findings were selected^9^,^13^,^15^,^16^; and (iii) conformations with the lowest binding energy and greatest number of hydrogen bonds were selected. Before exploring the interfaces, the final complexes were subjected to molecular dynamics simulations for stabilization and optimization. The Protein Interfaces, Surfaces, and Assemblies server (PISA)^56^ was used to calculate the buried surface interaction.

### Molecular dynamics simulation

GROMACSv4.6.2 was used to perform dynamics simulations^57^ for all final and optimized complexes after protein-protein docking under AMBER99SB-ILDN^58^ force field parameter sets. All complexes were solvated in a cubic box with a TIP3P water model^59^, and periodic boundary conditions were applied. To neutralize the system before production, Na^+1^ and Cl^−1^ ions were added where needed. Neutralized systems were subjected to energy minimization with a tolerance of 1,000 kJ/mol without applying any constraints, using the steepest descent integrator to remove any unfavorable interactions. The energy-minimized systems were subjected to two-step equilibration to obtain the starting structures for the production phase. To avoid any conformational changes, position restraints were applied to all atoms during the equilibration phases. First, under a constant volume (NVT) ensemble, the systems were simulated for 100 ps. The proteins and solvent with ions were treated as separate groups for temperature-coupling, using V-rescale method^60^. The equilibrated structures were equilibrated again at constant pressure (NPT; 1.0 bar)^61^. To constrain all bonds, the LINCS algorithm was used^62^. Finally, a 40 ns production run was performed under NPT conditions for protein-protein complexes and a 100 ns production run was performed for protein-ligand complexes. In protein ligand complex, the ligand topology was created with PRODRG^63^ online server after its charges were calculated with MOE. For protein-ligand simulations, GROMOS96 53A6^64^ force field was used. All production simulations were performed with a 2 fs time step, and the coordinates were saved every 2 ps under constant pressure (1 bar) and temperature (300 K) without any position restraints. The average structures extracted over the last 10 ns for all complexes were energy minimized to remove the stereo clashes and further used for interface analysis and molecular docking studies. The least square fit method was applied to calculate RMSDs and RMSFs for the final saved trajectories. Chimera 1.9^65^ and MOE 2013 platforms were used for all visualizations.

### Principal component analysis (PCA)

The combined movements of the molecules were determined by a set of eigenvectors and eigenvalues extracted from the covariance matrix diagonalization. The least-squares fit superimposition on the average structure for all complexes was used to remove the overall rotational and translational movements in the MD trajectory before PCA analysis. The g_covar package within GROMACS was used to calculate and diagonalize the covariance matrix for the coordinates of each complex and 60 frames were extracted for each trajectory to create a movie for visual inspection of domain movements. The g_anaeig was used to analyze and plot the eigenvectors of each complex.

### Protein-ligand docking

To identify the possible binding patterns of potent HPV E6 inhibitors, structures of already reported small molecules were built in MOE builder and ChemSketch (Advanced Chemistry Development, Inc., Toronto, Canada). Seventeen out of the total number of potential small molecules were selected from the literature^45^,^46^, and three were downloaded from the PubChem server (Table S7). The conformation search in MOE was used to calculate the 30 low-energy conformers for each ligand using a stochastic method under the MMFF94x force field with a 0.005 RMS gradient and were saved as a ligand database^66^. After global docking (without selecting any pocket in E6) using the Triangle Matcher placement method, the London dG scoring function was applied to rank the resulting docked conformations. The ASE Rescoring function was used to filter the five best-docked poses, retained for each conformer after refining by energy minimization in the pocket. A separate database with the 20 top-ranked poses, based on the docking scores, binding energies, and binding affinities, was filtered out of all docked conformers. The scoring function MM/GBVI estimates the binding free energy of the ligand for a given pose, whereas lower scores indicate more favorable poses^67^. The generalized Born/volume integral (GB/VI) implicit solvent method was used to determine the binding affinities of the potential ligands while keeping residues away from the ligand as rigid and receptor atoms in the vicinity of the ligand as well as the ligand itself as flexible. Every top-ranked pose was energy minimized in the binding pocket prior to calculation of the binding affinity.

### Alanine and resistance scanning of E6

Changes in the affinity of E6 pocket toward the ligand and stabilities of the E6-ligand complexes were determined through an alanine scan, distributed in the MOE suite under the protein design package. The top-ranked complexes were subjected to alanine scanning using Unary Quadratic Optimization (UQO) under the LowMode ensemble, which uses the LowModeMD^68^ to search the conformational space of the mutants. The LowModeMD search method generated mutant conformations using a short approximately 1 ps run of MD at constant temperature^69^ followed by an all-atom energy minimization under an MMFF94x force field. The resulting conformations were saved to the output database, when they satisfied the required conditions for energetics and geometrics criteria. To speed up the simulation, atoms farther than 4.5 Å were marked as inert, iterations were limited to 50, and conformations were limited to five for each mutated complex. Three residues, Leu50, Arg102, and Arg131, were mutated into alanine in each ligand-bound E6 complex and changes in binding affinity and stability of the complex were recorded. The same protocol was used for resistance scanning and to identify point mutations (SNPs) in E6, which might cause a loss or reduction in affinity toward the tested ligands.

## Additional Information

**How to cite this article**: Shah, M. *et al.* *In silico* mechanistic analysis of IRF3 inactivation and high-risk HPV E6 species-dependent drug response. *Sci. Rep.* **5**, 13446; doi: 10.1038/srep13446 (2015).

## Supplementary Material

Supplementary Information

Supplementary Video 1

Supplementary Video 2

Supplementary Video 3

Supplementary Video 4

Supplementary Video 5

## Figures and Tables

**Table 1:** Interface analysis of docked complexes before and after molecular dynamics (MD) simulation compared with the interface of the E6AP-E6 active crystal structure.

Complex	Status	Residues present at the interface of E6AP-E6 complex	ISA*, Å^2^
E6AP(4GIZ)	Before MD	E46 Q50 A53 T54 R67 K180 S338 A339 Y342 A343 T346 T367 N368 A371 E372 T374 **L375 Q376 E377 L378 L379** G380 E381 E382 R383	1272,
	After MD	E46 P49 Q50 A53 T54 Q73 S74 G75 L76 N174 K176 K180 P335 Q336 S338 A339 Y342 A371 E372 **T374 L375 Q376 E377 L378** L379 G380 E381 E382	1449,
E6	Before MD	R8 **R10 V31 Y32** L50 **C51 V53 R55** V62 L67 **Y70** S71 I73 S74 R77 H78 S80 Y81 Q91 Y92 L100 **R102** Q107 R129 G130 **R131**	1216,
	After MD	Q6 R10 K11 V31 Y32 D49 L50 **C51** V53 **R55** V62 L67 Y70 S71 I73 S74 **R77** H78 S80 Y81 L83 T87 Q90 Q91 Y92 L100 **R102** Q107 R129 G130 **R131**	1337,
ZDOCK	Before MD	E46 P49 Q50 A53 T54 R67 S74 K180 S338 A339 Y342 A343 T346 T367 N368 A371 E372 T374 **L375 Q376 E377 L378 L379** G380 E381 E382 R383	1478,
E6AP	After MD	E46 P49 Q50 A53 T54 R67 Q73 S74 L76 K180 P335 S338 A339 Y342 A370 A371 E372 T374 L375 **Q376 E377 L378 L379** G380 E381 E382	1321,
E6	Before MD	**R10** K11 **V31 Y32** D49 L50 **C51 V53 R55 V62** L67 **Y70** S71 I73 S74 R77 × 78 S80 Y81 Q91 Y92 L100 **R102** Q107 N127 R129 G130 **R131**	1340,
	After MD	R10 V31 Y32 L50 **C51** V53 **R55** V62 L67 Y70 S71 S74 R77 H78 S80 Y81 S82 L83 T87 Q90 Q91 Y92 L100 **R102** Q107 I128 **R129** G130 **R131**	1267,
		Residues present at the interface of the IRF3-E6 complex	
ZDOCK IRF3-LR2	Before MD	E189 N190 P191 K193 **R194** L196 V197 P198 E200 E203 E205 Q217 Q218 T219 S221 R236 W241 T370 R373 A374 E377 V381 S385 L387 E388 N389 T390 D392 H394 I395 **S396 N397 S398** H399 P400 L401 S402 L403 Q407 Y411	1661,
	After MD	**E189** K193 R194 V197 P198 **E200** E203 E205 Q217 Q218 T219 E232 T370 R373 A374 E377 V381 N389 T390 **D392 H394 N397 S398 H399** P400 S402	1234,
E6	Before MD	P5 Q6 **R8 R10** K11 V31 Y32 K34 D49 L50 **C51** I52 **V53** Y54 **R55** D56 G57 L67 F69 **Y70** I73 S74 R77 H78 S80 Y81 Q91 Y92 N93 K94 L100 R102 Q107 N127 I128 R129 G130 **R131**	1708,
	After MD	P5 Q6 **R8 R10** K11 Q14 **C51** V53 Y54 V62 F69 Y70 I73 S74 **R77** H78 S80 Y81 **Q91** Y92 N93 Q107 N127 I128 **R129** G130 **R131**	1233,
ZDOCK IRF3-LR1	Before MD	**S13** T15 **Q16 E17** D18 **I19** L20 **D21 E22 L23 L24 G25** N26	830
	After MD	**S13** D14 **T15** Q16 E17 **D18 I19 L20 D21 E22 L23 L24 G25 N26**	735
E6	Before MD	**R10 V31 Y32 L50** C51 **V53 R55 V62** L67 **Y70** S71 I73 **S74 R77 H78** R102 Q107 **R131**	900
	After MD	**R8 R10 V31** Y32 K34 **L50** C51 **V53 R55** D56 **V62 L67** Y70 S71 S74 R102 Q107 **R129 R131**	859

^*^Change in the surface area at the interface of the corresponding chains. Bold residues are involved in hydrogen bonding and hydrophobic interactions.

**Table 2:** Selected potent HPV E6 inhibitors with their predicted binding affinities and docking scores.

		IC50 nM	Dock-score (kcal/mol)	TPSA	Solvation (kcal/mol)	Affinity	
Compound ID						GBVI/WSA dG (kcal/mol)	London dG pKi (M)
1	Kaemferol^a^	20	−5.33	107.2	−32.51	−5.76	7.67
2	Morin^a^	4	−5.35	127.4	−35.90	−6.09	8.80
3	Myricetin^a^	0.85	−5.58	147.7	−34.44	−6.03	6.74
4	Luteolin^a^	23	−5.52	107.2	−33.50	−5.76	5.89
5	Caf24^b^	5.2	−5.55	89.99	−37.71	−6.57	6.16
6	Caf25^b^	1.1	−5.75	80.76	−37.53	−6.32	6.73
7	Caf26^b^	8.1	−5.38	80.76	−34.63	−6.19	6.21
8	Caf27^b^	6.9	−6.28	80.76	−44.04	−7.44	7.50
9	Caf28^b^	5.2	−5.42	83.83	−35.82	−6.18	6.61
10	Caf29^b^	12.5	−6.03	82.06	−40.12	−6.57	6.53
11	Caf30^b^	47.3	−5.56	63.60	−36.28	−6.56	7.16
12	Caf31^b^	62.2	−6.04	63.60	−38.04	−6.72	6.44
13	Caf32^b^	48	−5.89	63.60	−38.72	−6.34	7.11
14	SA s327301	52	−8.18	124.7	−54.13	−8.26	7.63
15	SA 207721	21	−6.54	116.0	−55.35	−8.38	7.14
16	SA r218634	27	−6.37	74.60	−37.18	−6.41	7.17
17	SA r225975	12	−6.42	91.60	−32.93	−5.92	7.21
18	SA r278319	17	−7.36	115.1	−57.84	−8.88	8.03
19	SA s204102	11	−7.78	158.4	−52.76	−8.43	7.37
20	NC 135098	22	−8.25	265.4	−59.11	−8.20	7.90

^a^Derivatives of 5,7-dihydroxy-4H-chromen-4-one (Kaemferol, Morin, and Myricetin were downloaded from PubChem).

^b^Derivatives of 4H-chromen-4-one were adopted form Reference 46. SA (Sigma Aldrich) and NCI (National Cancer Institute) chemicals selected from Reference 45. TPSA; Topological polar surface area. GBVI/WSA; Generalized born volume integral/Weighted surface area scoring function.
