# Supplementary material for: In silico mechanistic analysis of IRF3 inactivation and high-risk HPV E6 species-dependent drug response
Source: Sci Rep. 2015 Aug 20;5:13446. doi: 10.1038/srep13446 (PMC4542336; doi:10.1038/srep13446)
Supplement: Supplementary Information [file srep13446-s1.pdf]

Supplementary Information

In silico mechanistic analysis of IRF3 inactivation and high-risk HPV E6 species-dependent drug response

Masaud Shah[1], Muhammad Ayaz Anwar[1], Seolhee Park[1], Syyada Samra Jafri[2] & Sangdun Choi[1*]

[1]Department of Molecular Science and Technology, Ajou University, Suwon, 443-749, Korea
[2]The Center of Excellence in Molecular Biology, University of the Punjab, Lahore, 54890, Pakistan

Correspondence and requests for materials should be addressed to

S.C. (sangdunchoi@ajou.ac.kr)

Sangdun Choi

Department of Molecular Science and Technology, Ajou University, Suwon 443-749, Korea

Phone: +82-31-219-2600; Fax: +82-31-219-1615

E-mail: sangdunchoi@ajou.ac.kr

Figure S1 | Structural stability of different complexes. (A) Root mean squares deviation (RMSD) plots calculated for the backbone atoms showing the stability of the docked complexes during molecular dynamics simulation with reference to the active crystal of MBP-E6AP-E6 (4GIZ). (B) Distance plots between the centers of masses of partner proteins in each complex along the entire production run. (C) The root mean squares fluctuation of the atoms in each complex. Extreme peaks represent the terminal loops.

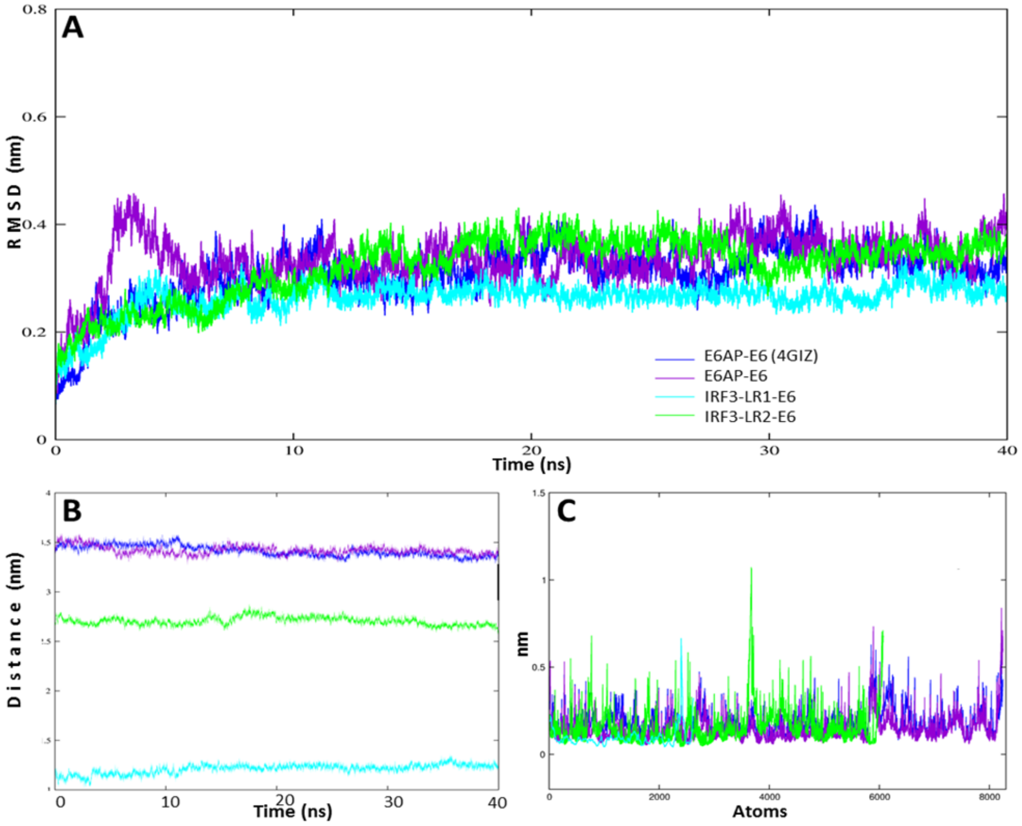

Figure S2 | RMSD and RMSF plots calculated for ligand-bound HPV 16 E6 protein. (A) Stable RMSD plot calculated for the backbone atoms of the ligand-bound E6 complex. (B) The distance between the ligand and E6 fluctuate in the first 50 ns of the simulation because of the hydrophobic tail of the ligand, as indicated by the RMSF (high peak). (C) The root mean squares fluctuations of all atoms in the complexes. All receptor atoms remain stable throughout the simulation except for those in the hydrophobic tail of the ligand, which has relatively high flexibility. Aromatic rings in the ligand remain stable (red circle). RMSD, root mean squares deviation; RMSF, RMS fluctuation.

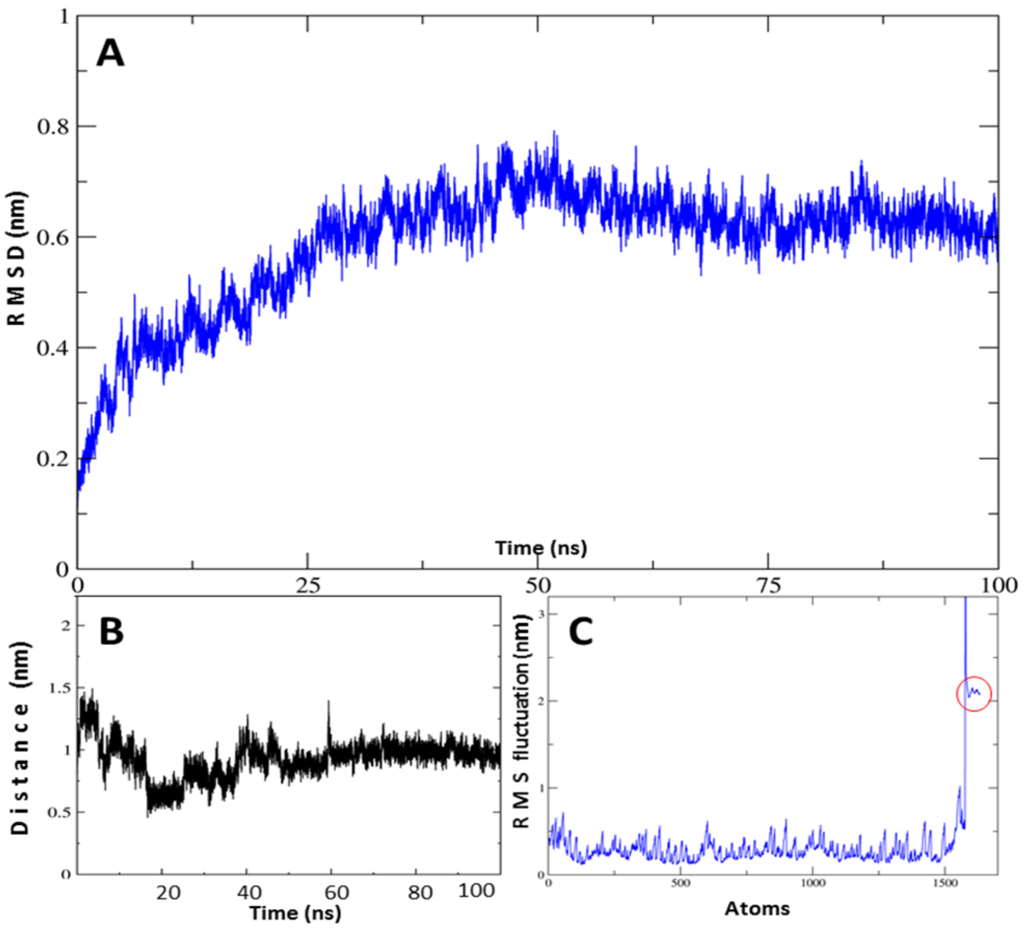

Figure S3 | Computational mutagenesis and alanine scanning. The binding free energy differences of the hot spot residues in E6AP-E6 and IRF3-E6 complexes were calculated when the wild-type residue was mutated to alanine. The potential hot spot residues are indicated as positive high binding free energy differences.

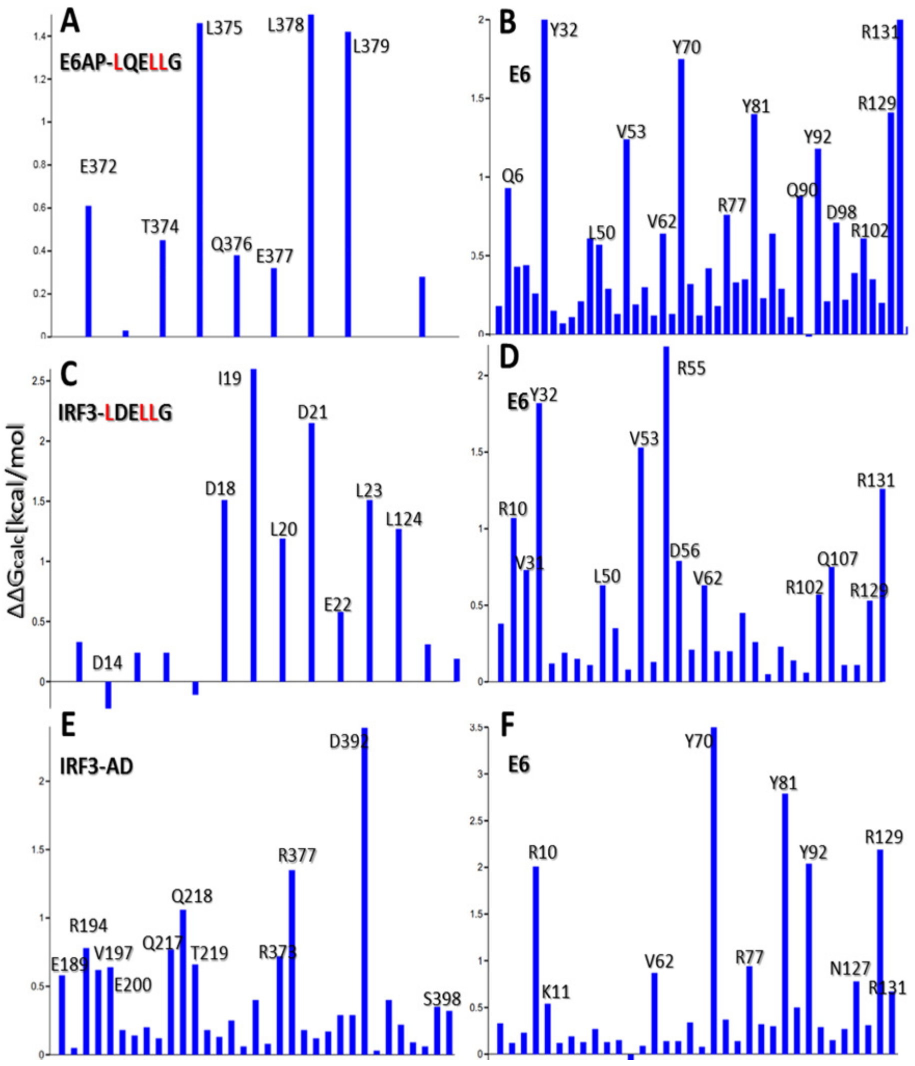

Figure S4 | The relative binding affinity (daffinity) and thermostability (dstability) of the mutant to the wild type protein. A more negative value indicates a mutation with better affinity and stability. Positive value indicate that wiled type residues is importanst for the stability of the complex. The units of daffinity and dstability are given in kcal/mol.

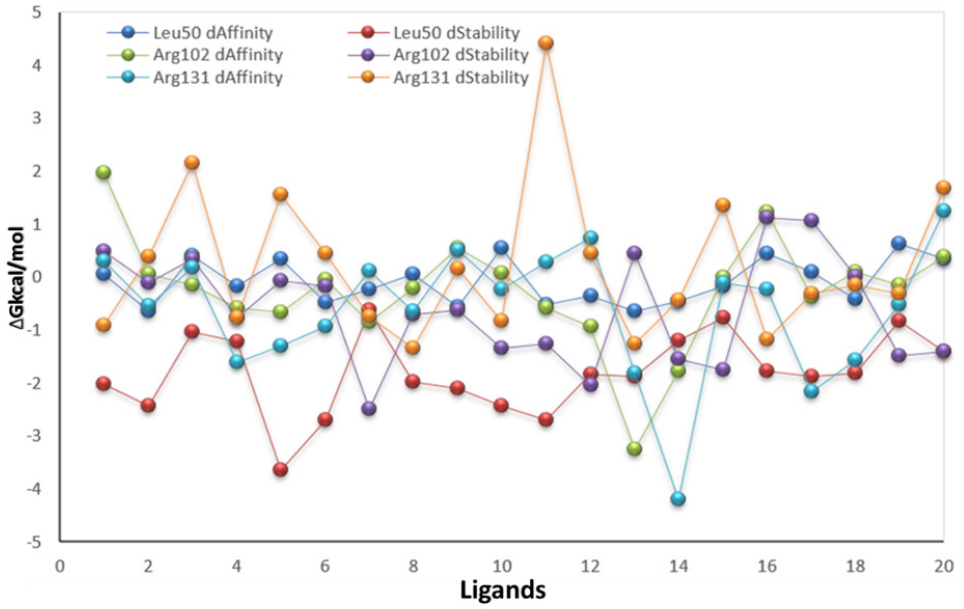

Figure S5 | Multiple sequence alignment of high-risk HPV E6 protein with reference to the crystal structure of E6. Alignment of the E6 sequences from five representative high-risk HPV genotypes. The positions that exhibit conservation among all high-risk HPV E6 are highlighted in light green, while those conserved in genotypes 16 and 18 only are highlighted in light pink. The secondary structure at the bottom of alignment corresponds to the active crystal structure of E6 (4GIZ). The red asterisks represent residues that are involved in E6AP and IRF3 binding. Residues are numbered according to the crystal structure of E6 (4GIZ) having one missing residue in its N-terminal.

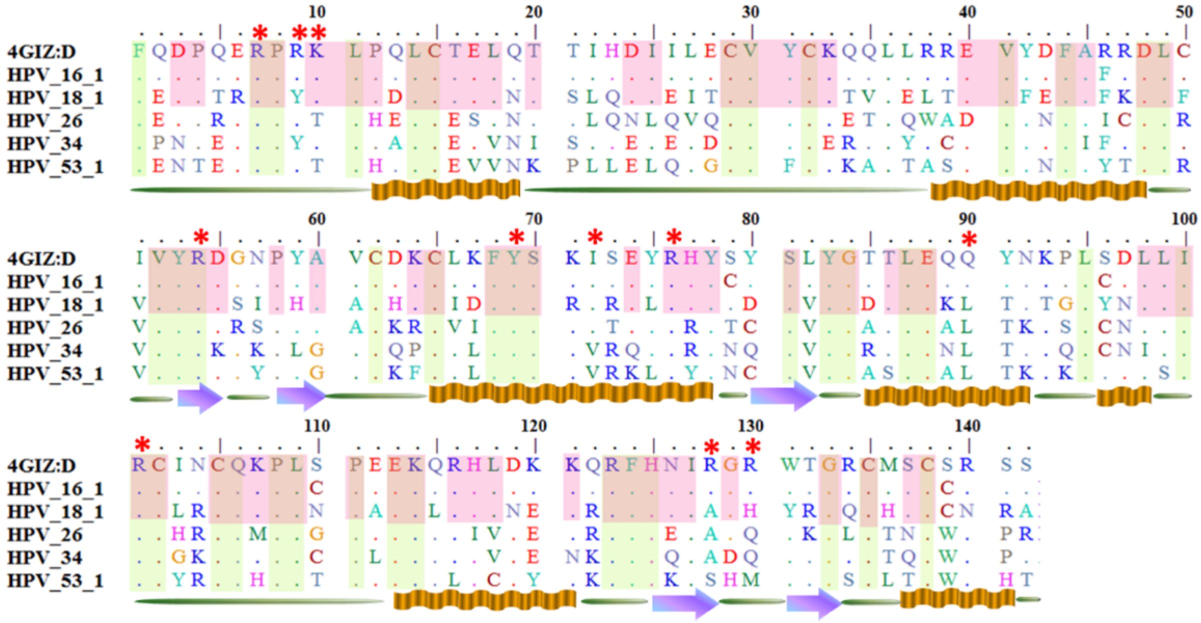

Table S1 | Hydrogen (HB) and hydrophobic (HYD) bonds present at the interface of the E6 and E6AP (PDB crystal) complex.

No.	Bond type	E6AP	HPV E6
1	HB	SER74.OG	TYR81.OH
5	HB	THR374.OG1	ARG55.NH2
3	HB	GLN376.OE1	ARG131.NE
4	HB	GLU377.OE2	ARG55.NH2
5	HB	GLY380.O	ARG131.NH2
6	HYD	LEU375.CD1	LEU67.CD2
7	HYD	LEU378.CD1	VAL31.CG1
8	HYD	LEU378.CD2	PHE45.CE1
9	HYD	LEU378.CD2	VAL53.CG2
10	HYD	LEU378.CD1	VAL62.CB
11	HYD	LEU379.CD2	LEU50.CD2

Table S2 | Hydrogen (HB) and hydrophobic (HYD) bonds present at the interface of the E6 and E6AP docked complex.

No.	Bond type	E6AP (4GIZ)	HPV E6 (4GIZ)
1	HB	SER74.OG	TYR81.OH
3	HB	GLU372.OE2	ARG77.NH1
4	HB	GLU372.OE1	HIS78.NE2
5	HB	THR374.OG1	ARG55.NH2
6	HB	GLU377.OE1	ARG55.NH2
7	HB	GLY380.O	ARG131.NH2
8	HB	GLU382.OE1	LYS11.NZ
9	HB	GLU382.OE2	ARG102.NH2
10	HB	ARG383.NH2	ASP98.OD1
11	HYD	LEU375.CD1	LEU67.CD1
12	HYD	LEU378.CD1	VAL31.CG1
13	HYD	LEU378.CD2	PHE45.CE1
14	HYD	LEU378.CG	VAL53.CG1
15	HYD	LEU378.CD1	VAL62.CB
16	HYD	LEU379.CD2	LEU50.CD2

Table S3 | Hydrogen (HB) and hydrophobic (HYD) bonds present at the interface of the E6 and IRF3-LR1 (leucine-rich region 1) complex.

No.	Bond type	HPV E6	IRF3-LR1
1	HB	ARG8.NH2	ASN26.OD1
2	HB	ARG10.NH1	ASN26.O
3	HB	ARG10.NH1	MET27.OC2
4	HB	ILE52.O	ASN26.ND2
5	HB	ARG55.NH1	SER13.OG
6	HB	ARG55.NE	THR15.O
7	HB	ARG55.NH2	GLU22.OE2
8	HB	ARG129.NH1	ASP18.OD1
9	HB	ARG131.NE	ASP21.OD1
10	HYD	VAL31.CG2	ILE19.CD1
11	HYD	VAL31.CG1	LEU23.CD1
12	HYD	LEU50.CD2	LEU24.CD2
13	HYD	VAL53.CG1	ILE19.CG2
14	HYD	VAL53.CG1	LEU23.CG
15	HYD	VAL62.CG1	LEU23.CD2
16	HYD	LEU67.CD1	LEU20.CD1

Table S4 | Hydrogen (HB) and hydrophobic (HYD) bonds present at the interface of the E6 and IRF3-LR2 complex.

No.	Bond type	HPV E6	IRF3-LR2
1	HB	ARG8.NH2	GLU189.OE1
2	HB	GLN91.O	GLN218.NE2
3	HB	TYR81.OH	THR219.N
4	HB	TYR92.OH	THR219.O
5	HB	ASN127.ND2	THR219.OG1
6	HB	ARG129.O	THR370.OG1
7	HB	ARG77.NE	GLU377.OE2
8	HB	ARG129.NH2	GLU377.OE1
9	HB	ARG77.NH2	THR390.O
10	HB	ARG131.NH1	ASP392.OD2
11	HB	ARG131.NE	HIS394.O
12	HB	LYS11.NZ	ASN397.OD1
13	HB	ARG131.NH2	SER398.OG

Table S5 | Relative binding affinities (daffinity) and thermostabilities (dstability) of the mutants to the wild type protein. A more negative value indicates a mutation with better affinity. The units of daffinity and dstability are given in kcal/mol.

		Leu50		Arg102		Arg131	
	Ligands	dAffinity	dStability	dAffinity	dStability	dAffinity	dStability
1	Kaemferol[a]	0.0511	-2.0295	1.9606	0.4719	0.3033	-0.9262
2	Morin[a]	-0.6536	-2.4346	0.0407	-0.1163	-0.5429	0.3823
3	Myricetin[a]	0.3947	-1.0389	-0.1645	0.3359	0.1648	2.145
4	Luteolin[b]	-0.1695	-1.2306	-0.5967	-0.7758	-1.6251	-0.7764
5	Caf24[b]	0.3282	-3.6559	-0.6718	-0.0736	-1.3066	1.555
6	Caf25[b]	-0.4944	-2.7097	-0.0509	-0.1849	-0.9406	0.4471
7	Caf26[b]	-0.2457	-0.6291	-0.8485	-2.4994	0.1195	-0.7462
8	Caf27[b]	0.0385	-1.9908	-0.2111	-0.7053	-0.654	-1.3542
9	Caf28[b]	-0.5732	-2.1004	0.5519	-0.6398	0.4992	0.1443
10	Caf29[b]	0.5352	-2.4358	0.0692	-1.3399	-0.2373	-0.8287
11	Caf30[b]	-0.5344	-2.7147	-0.5982	-1.2611	0.2682	4.4101
12	Caf31[b]	-0.3646	-1.845	-0.9391	-2.0508	0.7278	0.4445
13	Caf32[b]	-0.644	-1.8731	-3.2501	0.4294	-1.8116	-1.262
14	SA s327301	-0.4554	-1.1974	-1.7801	-1.5569	-4.206	-0.4493
15	SA 207721	-0.1676	-0.7678	-0.0038	-1.7693	-0.1177	1.3507
16	SA r218634	0.4402	-1.7734	1.2267	1.1242	-0.2477	-1.1758
17	SA r225975	0.0801	-1.885	-0.3779	1.0572	-2.1693	-0.3299
18	SA r278319	-0.4264	-1.8237	0.0805	0.0025	-1.5804	-0.1672
19	SA s204102	0.6178	-0.8325	-0.1478	-1.4956	-0.5182	-0.3249
20	NC 135098	0.3328	-1.4209	0.3718	-1.4108	1.2312	1.6716

[a]Derivatives of 5,7-dihydroxy-4H-chromen-4-one (Kaemferol, Morin, and Myricetin were downloaded from PubChem). [b]Derivatives of 4H-chromen-4-one were selected from the literature. SA (Sigma Aldrich) and NCI (National Cancer Institute) chemical, selected from the literature.

Table S6 | Relative binding affinities (daffinity) of proteins with single nucleotide polymorphisms (SNPs) to the wild type protein. Large positive increases in affinity indicate that the target might easily become resistant to the ligand if the respective SNP occurred. The units of daffinity are given in kcal/mol.

	Mutation	dAffinity
1	1:R131H	2.1041
2	1:R131S	2.0901
3	1:R102S	1.8451
4	1:R102G	1.5610
5	1:R131I	1.5549
6	1:R102N	1.5545
7	1:R131W	1.2831
8	1:R131N	1.2049
9	1:R131P	0.8213
10	1:R102I	0.8115
11	1:L50R	0.7445
12	1:R131K	0.3591
13	1:R102K	0.3149
14	1:L50P	0.3044
15	1:R102Q	0.2782
16	1:R131L	0.2680
17	1:L50V	0.2178
18	1:R102L	0.1609
19	1:R102M	0.1466
20	1:L50Q	0.0983
21	1:R131Q	0.0946
22	1:R131T	0.0574
23	1:R131M	0.0288
24	1:R102H	0.0171

Table S7 | Reported ligands depicted and colored according to their heteroatoms. [a]Derivatives of 5,7-dihydroxy-4H-chromen-4-one (Kaemferol, Morin, and Myricetin were downloaded from PubChem). [b]Derivatives of 4H-chromen-4-one were selected from the literature. SA (Sigma Aldrich) and NCI (National Cancer Institute) chemical, selected from the literature.

ID	Structure	ID	Structure
Luteolin[a]	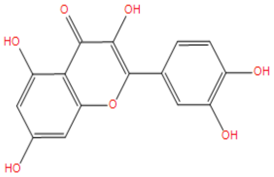	Caf24[b]	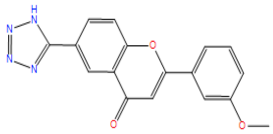
Kaemferol[a]	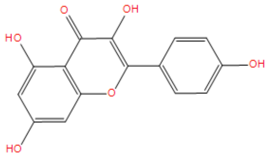	Caf25 [b]	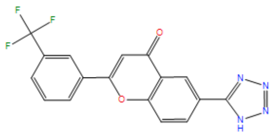
Morin[a]	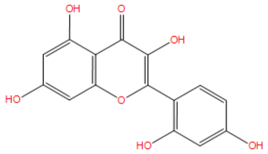	Caf26 [b]	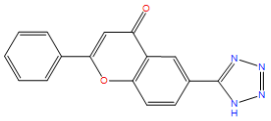
Myricetin[a]	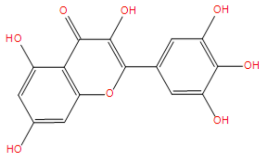	Caf27 [b]	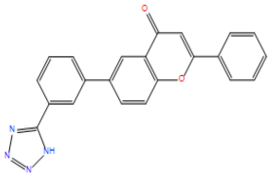
SA 327301	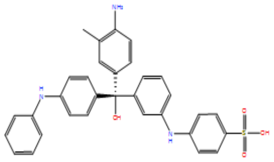	Caf28 [b]	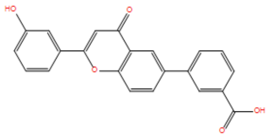

SA 207721	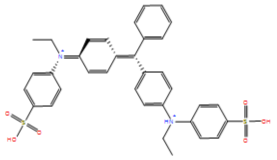	Caf29 [b]	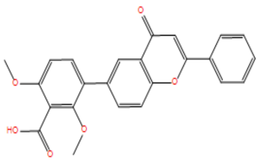
SA 218634	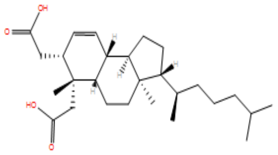	Caf30 [b]	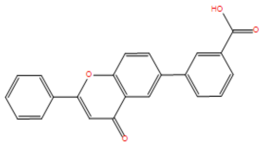
SA 278319	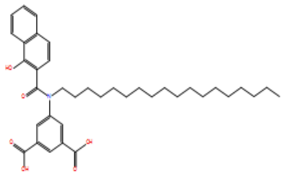	Caf31 [b]	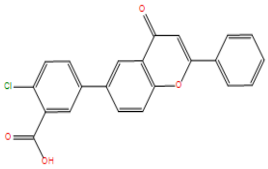
SA 204102	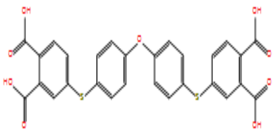	Caf32 [b]	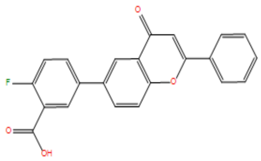
SA 225975	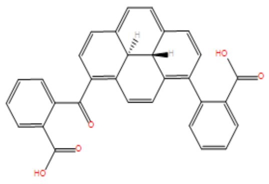	NC 135098	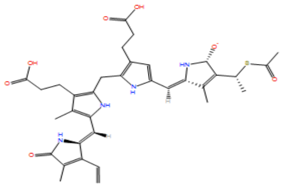

Video S1 | Principal movements of the domains in E6AP-E6 (4GIZ) complex over 40 ns molecular dynamics simulation (MDS). Prominent outward movements of the two zinc binding domains of E6 were detected when LxxLL motif of E6AP was bound into the E6 pocket.

Video S2 | Movements of different domains in the E6AP-E6 (protein-protein docked) complex over 40 ns molecular dynamics simulation (MDS).Similar tendency of the hydrophobic leucine residues in LxxLL motif of E6AP towards the pocket of E6 was observed as depicted in video S1.

Video S3 | Similar domain-dynamics of E6, when bound to the LxxLL motif of E6AP and IRF3-LR1, confirmed the possible molecular interaction of E6 with LR1 of IRF3. The fitting of IRF3-LR1 in E6 pocket resembles to that of the E6AP-E6 crystal complex.

Video S4 | Transient shifting of E6 from IRF3-LR2 (Arg194) towards the autoinhibitory domain (AD) domain (Ser396 and Ser398) of IRF3 can be seen, suggesting to the possible masking of AD and phosphorylation inactivation of IRF3 by E6.

Video S5 | Ligand binding pocket of E6. Rigorous movement of E6 pocket was observed during molecular dynamics (MD) run, indicating the possible anti-E6 inhibitory activity of the ligand.
